# Synthesis of High Molecular Weight Water-Soluble Polymers as Low-Viscosity Latex Particles by RAFT Aqueous Dispersion Polymerization in Highly Salty Media

**DOI:** 10.1021/acs.macromol.2c01071

**Published:** 2022-08-28

**Authors:** Rory J. McBride, John F. Miller, Adam Blanazs, Hans-Joachim Hähnle, Steven P. Armes

**Affiliations:** † Chemistry Department, University of Sheffield, Brook Hill, Sheffield, South Yorkshire S3 7HF, U.K.; ‡ Enlighten Scientific LLC, Hillsborough, North Carolina 27278, United States; § BASF SE, RAM/OB - B001, Carl-Bosch-Strasse 38, 67056 Ludwigshafen am Rhein, Germany

## Abstract

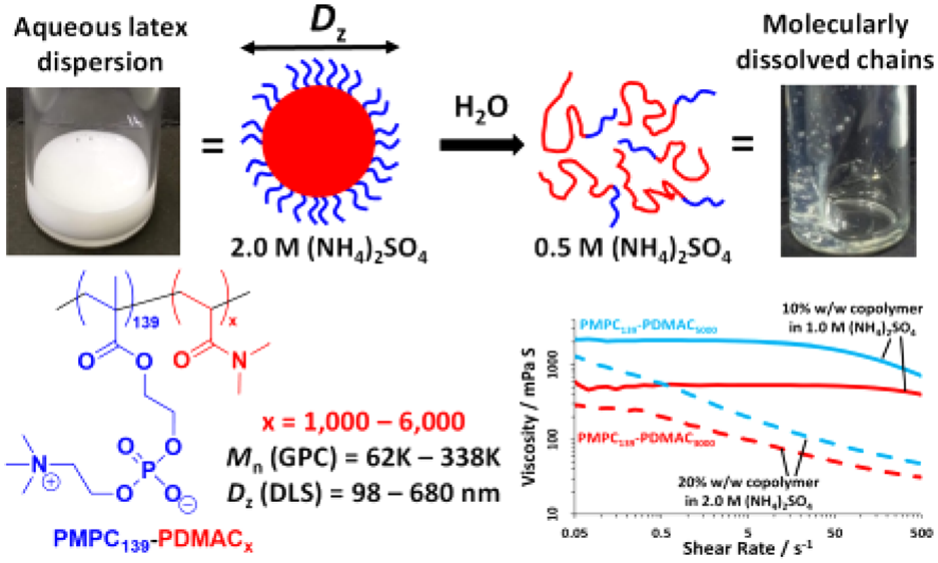

We report the synthesis of sterically-stabilized diblock copolymer particles at 20% w/w solids via reversible addition–fragmentation chain transfer (RAFT) aqueous dispersion polymerization of *N*,*N*′-dimethylacrylamide (DMAC) in highly salty media (2.0 M (NH_4_)_2_SO_4_). This is achieved by selecting a well-known zwitterionic water-soluble polymer, poly(2-(methacryloyloxy)ethyl phosphorylcholine) (PMPC), to act as the salt-tolerant soluble precursor block. A relatively high degree of polymerization (DP) can be targeted for the salt-insoluble PDMAC block, which leads to the formation of a turbid free-flowing dispersion of PDMAC-core particles by a steric stabilization mechanism. ^1^H NMR spectroscopy studies indicate that relatively high DMAC conversions (>99%) can be achieved within a few hours at 30 °C. Aqueous GPC analysis indicates high blocking efficiencies and unimodal molecular weight distributions, although dispersities increase monotonically as higher degrees of polymerization (DPs) are targeted for the PDMAC block. Particle characterization techniques include dynamic light scattering (DLS) and electrophoretic light scattering (ELS) using a state-of-the-art instrument that enables accurate ζ potential measurements in a concentrated salt solution. ^1^H NMR spectroscopy studies confirm that dilution of the as-synthesized dispersions using deionized water lowers the background salt concentration and hence causes in situ molecular dissolution of the salt-intolerant PDMAC chains, which leads to a substantial thickening effect and the formation of transparent gels. Thus, this new polymerization-induced self-assembly (PISA) formulation enables high molecular weight water-soluble polymers to be prepared in a highly convenient, low-viscosity form. In principle, such aqueous PISA formulations are highly attractive: there are various commercial applications for high molecular weight water-soluble polymers, while the well-known negative aspects of using a RAFT agent (i.e., its cost, color, and malodor) are minimized when targeting such high DPs.

## Introduction

It is well-known that reversible addition-fragmentation chain transfer (RAFT) polymerization enables the synthesis of a wide range of functional vinyl polymers with good control over the molecular weight distribution.^1−4^ There are many literature examples of RAFT solution homopolymerization and, in some cases, mean degrees of polymerization (DP) up to (and even beyond) 10,000 have been targeted.^5−7^ This latter aspect is interesting for two reasons. First, in the case of water-soluble polymers (e.g., polyacrylamide), such high molecular weights are useful for commercial applications such as flocculants, binders, or thickeners.^8−10^ Second, the main disadvantage of RAFT chemistry is that the chain transfer agent is an organosulfur compound, which is relatively expensive and confers both malodor and color.^11^ Since the mean DP is inversely proportional to the concentration of this RAFT agent,^12^ targeting very high DPs minimizes the problems associated with its use, which could make a decisive difference to the feasibility of industrial scale-up.^13^

However, the synthesis of high molecular weight water-soluble polymers via RAFT aqueous solution polymerization leads to extremely viscous reaction mixtures. For example, gel formation was reported by Destarac and co-workers when preparing polyacrylamide with a mean DP of around 10,000.^6^ Such gels can be difficult to remove from the reaction vessel after the polymerization, and heat dissipation during polymerization can become inefficient. In principle, this problem could be addressed by conducting such polymerizations in highly salty media. Under such conditions, the water-soluble polymer chains become insoluble, which leads to the formation of low-viscosity, free-flowing particulate dispersions rather than highly viscous or gel-like aqueous solutions.^14^ Indeed, this approach is used to prepare high molecular weight polyacrylamide in the form of particles via conventional free radical polymerization conducted in aqueous solution in the presence of 2.0 M ammonium sulfate.^15−17^

**Scheme 1:**
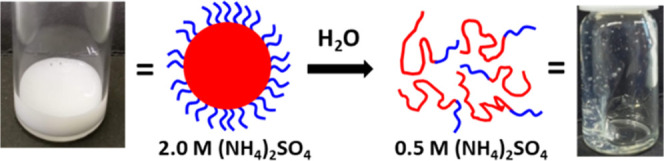
Schematic Cartoon and Corresponding Digital Images to Illustrate the Sterically Stabilized Diblock Copolymer Particles in the Presence of 2.0 M Ammonium Sulfate Obtained after RAFT Aqueous Dispersion Polymerization of a Suitable Water-Soluble Monomer to Form the “Salted Out” Red Chains A four-fold dilution with deionized water lowers the salt concentration of the initial aqueous dispersion and results in molecular dissolution of these particles, with the concomitant formation of a highly viscous transparent aqueous solution.

Polymerization-induced self-assembly (PISA) involves the growth of an insoluble block from a soluble precursor block in a suitable solvent. In the case of an aqueous PISA formulation, the growing second block becomes water-insoluble while the first block remains water-soluble: the resulting amphiphilic diblock copolymer chains undergo micellar nucleation and ultimately sterically-stabilized diblock copolymer nanoparticles are produced. Depending on the aqueous solubility of the monomer used to generate the hydrophobic block, aqueous PISA formulations can involve either RAFT aqueous emulsion polymerization^18−20^ or RAFT aqueous dispersion polymerization.^21−26^ In both cases, the hydrophobic block normally remains insoluble at the end of the polymerization. However, there are several literature examples in which a temperature switch leads to *in situ* nanoparticle dissolution to yield molecularly dissolved diblock copolymer chains.^27−29^ We hypothesized that a similar approach might involve the synthesis of a salt-intolerant water-soluble polymer in highly salty media to produce low-viscosity particles. Subsequent dilution using pure water would then lower the salt concentration in the aqueous continuous phase, which should lead to the molecular dissolution of the high molecular weight copolymer chains within the particle cores and hence a strong thickening effect (see Scheme 1).

According to well-established principles in colloid science, steric stabilization is much more likely to be effective than charge stabilization for such aqueous PISA syntheses.^30−32^ Clearly, such formulations would require a steric stabilizer that remains soluble in the presence of substantial amounts of salt to confer effective colloidal stabilization. According to the literature, suitable salt-tolerant water-soluble polymeric stabilizers are likely to be either certain types of polyelectrolytes^16,17,33,34^ or polybetaines.^35−37^

Herein, we report the RAFT aqueous dispersion polymerization of *N,N*′*-*dimethylacrylamide (DMAC) in highly salty media using poly(2-(methacryloyloxy)ethyl phosphorylcholine) (PMPC) as a salt-tolerant steric stabilizer. According to the literature, PMPC remains soluble even in the presence of 5.0 M NaCl.^35^ This approach is then extended to include polyelectrolytic steric stabilizers.

## Experimental Section

### Materials

2-(Methacryloyloxy)ethyl phosphorylcholine (MPC) was kindly donated by Biocompatibles (U.K.), and ammonium sulfate was purchased from Alfa Aesar (U.K.) and Eisen-Golden Laboratories (CA). 2-Ethylhexanoyl *tert*-butyl peroxide (T21S) was obtained from AkzoNobel (Netherlands), potassium hydroxide was obtained from LabChem (PA), and 2,2′-azobis(2-imidazolinylpropane) dihydrochloride (VA-044) was obtained from Fluorochem (U.K.). *N,N*′-Dimethylacrylamide (DMAC), ascorbic acid (AsAc), potassium persulfate (KPS), 4,4′-azobis(4-cyanopentanoic acid) (ACVA), azobis(isobutyl)amidine dihydrochloride (AIBA), CH_3_COOH, NaH_2_PO_4_, NaNO_3_, KOH, a 50% solution of 2-acrylamido-2-methyl-1-propanesulfonic acid sodium salt (AMPS), phosphate buffer solution (PBS) tablets, and D_2_O were purchased from Sigma-Aldrich (U.K.). Each of these chemicals was used as received.

2-(Acryloyloxy)ethyl trimethylammonium chloride (ATAC) was donated by BASF (Germany) in the form of an 80% w/w aqueous solution. 4-Cyano-4-(2-phenylethanesulfanylthiocarbonyl)-sulfanylpentanoic acid (PETTC) was prepared and purified as reported elsewhere,^38^ as was 2-(((butylthio)carbonothioyl)thio)-2-methylpropanoic acid (BDMAT).^39,40^ All solvents were purchased from Fisher Scientific (U.K.) and were used as received.

### Synthesis Protocols

#### Synthesis of the PMPC_139_ Precursor via RAFT Solution Polymerization of 2-(Methacryloyloxy)ethyl phosphorylcholine (MPC) in Methanol at 64 °C

PETTC (250 mg, 0.74 mmol), MPC (26.10 g, 88.4 mmol), ACVA (41 mg, 150 μmol), and methanol (49.0 g, corresponding to a 35% w/w solution) were weighed into a 250 mL round-bottom flask charged with a magnetic flea and this reaction solution was degassed using nitrogen gas for 45 min at 20 °C. The sealed flask was immersed into an oil bath set at 64 °C for 210 min, and the polymerization was subsequently quenched by exposing the reaction mixture to air while cooling to 20 °C. The final MPC conversion was 75%, as judged by ^1^H NMR spectroscopy (calculated by comparing the integrated vinyl signals assigned to the MPC monomer at 5.6–6.2 ppm to the integrated polymethacrylic backbone signals at 0.6–2.4 ppm). The reaction solution was precipitated into a ten-fold excess of acetone. The crude PMPC precursor was redissolved in methanol, and the precipitation was repeated. After dissolution using deionized water, the resulting aqueous polymer solution was freeze-dried overnight. The degree of polymerization was 135 ± 10, as judged by ^1^H NMR spectroscopy (calculated by comparing the integrated aromatic signals assigned to the RAFT end-group at 7.1–7.4 ppm to the integrated polymethacrylic backbone signals at 0.6–2.4 ppm). RAFT end-group analysis using UV spectroscopy indicated a mean degree of polymerization of 139 ± 1 (the Beer–Lambert plot for PETTC is provided in Figure S1). Aqueous GPC analysis indicated an *M*_n_ of 17 kg mol^–1^ and an *M*_w_/*M*_n_ of 1.18 (see below for eluent and calibration details).

#### Preparation of 2.0 M Ammonium Sulfate Solution and Redox Initiator Solutions

Ammonium sulfate (26.43 g) was added to a 100 mL volumetric flask, which was subsequently filled with water to obtain a 2.0 M solution. The required molarity, refractive index, and dynamic viscosity for an aqueous solution of 2.0 M ammonium sulfate were calculated by interpolation of tabulated solution properties reported at 20 °C as recorded in Table S1.^41^ The interpolated dynamic viscosity was estimated to 25 °C using the ratio of the dynamic viscosity for water at 20 and 25 °C. The following numerical values for a 2.0 M aqueous solution of ammonium sulfate were used in this study: molality = 2.32 mol kg^–1^; refractive index = 1.370, and dynamic viscosity = 1.367 × 10^–3^ kg m^–1^ s^–1^. The relative permittivity of a 2.0 M aqueous solution of ammonium sulfate was assumed to be that of pure water. According to the literature, the addition of salt leads to a lower relative permittivity compared to that of water.^42^ However, this systematic error is not considered to be important relative to the likely error incurred when calculating the ζ potential for electrosterically-stabilized nanoparticles^43^ (see Results and Discussion for further details).

KPS (30.0 mg) was dissolved in an aqueous solution of 2.0 M ammonium sulfate (30 g) to make up a 0.1% w/w KPS stock solution. Similarly, AsAc (30.0 mg) was dissolved in an aqueous solution of 2.0 M ammonium sulfate (30 g) to make up a 0.1% w/w AsAc stock solution. Each stock solution was stored in a refrigerator at 4 °C prior to use.

#### Synthesis of PMPC_139_–PDMAC*_x_* Diblock Copolymer Particles via RAFT Aqueous Dispersion Polymerization of *N*,*N*′-Dimethylacrylamide (DMAC) in 2 M Ammonium Sulfate at 30 °C

A typical protocol for the synthesis of PMPC_139_–PDMAC_5000_ spheres at 20% w/w solids was conducted as follows. The PMPC_139_ precursor (140 mg, 4.0 μmol), DMAC (1.972 g, 19.9 mmol), the 0.1% aqueous solution of KPS (270 mg, 1.0 μmol), and an aqueous solution of 2.0 M ammonium sulfate (8.00 g) were weighed into a 25 mL round-bottom flask charged with a magnetic flea and this reaction solution was degassed using nitrogen gas for 30 min at 20 °C. The sealed flask was immersed into an oil bath set at 30 °C, and a 0.1% aqueous solution of AsAc (170 mg, 1.0 μmol) was added to initiate the DMAC polymerization. After 18 h, the polymerization was subsequently quenched by exposing the reaction mixture to air while cooling to 20 °C. The final DMAC conversion was more than 99%, as judged by ^1^H NMR spectroscopy (as calculated by comparing the integrated vinyl signals assigned to the DMAC monomer at 5.6–6.7 ppm to the integrated polyacrylamide backbone signals at 1.1–2.7 ppm). Aqueous GPC analysis indicated an *M*_n_ of 262 kg mol^–1^ and an *M*_w_/*M*_n_ of 1.97 (see below for eluent and calibration details).

#### Synthesis of PATAC_195_–PDMAC_1000_ Diblock Copolymer Particles via RAFT Aqueous Dispersion Polymerization

PETTC (290 mg, 0.85 mmol), ATAC (80% w/w in water) (41.35 g, 170 mmol), AIBA (46 mg, 170 μmol), and methanol (50.1 g, corresponding to a 40% w/w solution) were weighed into a 250 mL round-bottom flask charged with a magnetic flea, and this reaction solution was degassed using nitrogen gas for 45 min at 20 °C. The sealed flask was immersed into an oil bath set at 56 °C for 120 min, and the polymerization was then quenched by exposing the reaction mixture to air while cooling to 20 °C. The final ATAC conversion was 97%, as judged by ^1^H NMR spectroscopy (calculated by comparing the integrated vinyl signals assigned to the ATAC monomer at 5.8–6.4 ppm to the integrated polyacrylic backbone signals at 1.3–2.7 ppm). Excess water was added, and the methanol was removed under reduced pressure. Afterward, the reaction solution was purified by dialysis over 3 days with regular water changes. The resulting aqueous polymer solution was freeze-dried overnight. The degree of polymerization was 195, as judged by ^1^H NMR spectroscopy (calculated by comparing the integrated aromatic signals assigned to the RAFT end-group at 7.1–7.4 ppm to the integrated polyacrylic backbone signals at 1.3–2.7 ppm). Aqueous GPC analysis indicated an *M*_n_ of 34 kg mol^–1^ and an *M*_w_/*M*_n_ of 1.20 (see below for eluent and calibration details).

Subsequently, the PATAC_195_ precursor (120 mg, 3.2 μmol), DMAC (312 mg, 3.15 mmol), a 0.1% aqueous solution of VA-044 (339 mg, 1.0 μmol), and an aqueous solution of 2.0 M sulfate (3.55 g) were weighed into a 10 mL round-bottom flask charged with a magnetic stirrer, and this reaction solution was degassed using nitrogen gas for 30 min at 20 °C. The sealed flask was immersed into an oil bath set at 48 °C to initiate the DMAC polymerization. After 18 h, the polymerization was quenched by exposing the reaction mixture to air while cooling to 20 °C. The final DMAC conversion was more than 99%, as judged by ^1^H NMR spectroscopy (as calculated by comparing the integrated vinyl signals assigned to the DMAC monomer at 5.6–6.7 ppm to the integrated polyacrylamide backbone signals at 1.1–2.7 ppm). Aqueous GPC analysis indicated an *M*_n_ of 68 kg mol^–1^ and an *M*_w_/*M*_n_ of 1.95 (see below for eluent and calibration details).

#### Synthesis of PAMPS_250_–PDMAC_1000_ Diblock Copolymer Particles via RAFT Aqueous Dispersion Polymerization

BDMAT (150 mg, 0.59 mmol), AMPS (55% w/w) (61.92 g, 149 mmol), T21S (25.7 mg, 119 μmol), and 1.0 M PBS (23.5 g, corresponding to a 40% w/w solution) were weighed into a 250 mL round-bottom flask charged with a magnetic flea, and this reaction solution was degassed using nitrogen gas for 45 min at 20 °C. The sealed flask was immersed into an oil bath set at 90 °C for 150 min and the polymerization was subsequently quenched by exposing the reaction mixture to air while cooling to 20 °C. The final AMPS conversion was 99% as judged by ^1^H NMR spectroscopy (calculated by comparing the integrated vinyl signals assigned to the AMPS monomer at 5.6–6.2 ppm to the integrated polyacrylic backbone signals at 1.2–2.3 ppm). The reaction solution was purified by dialysis against water for three days. The resulting aqueous polymer solution was freeze-dried overnight. The degree of polymerization was 250 as judged by ^1^H NMR spectroscopy (calculated by comparing the integrated methyl signals assigned to the RAFT end-group at 0.8–0.9 ppm to the integrated acrylic backbone signals at 1.2–2.3 ppm). Aqueous GPC analysis indicated an *M*_n_ of 28 kg mol^–1^ and an *M*_w_/*M*_n_ of 1.35 (see below for eluent and calibration details).

Subsequently, the PAMPS_250_ precursor (220 mg, 3.8 μmol), DMAC (379 mg, 3.82 mmol), a 0.1% aqueous solution of VA-044 (344 mg, 1.3 μmol), and an aqueous solution of 2.0 M (NH_4_)_2_SO_4_ (2.05 g) were weighed into a 10 mL round-bottom flask charged with a magnetic stirrer, and this reaction solution was degassed using nitrogen gas for 30 min at 20 °C. The sealed flask was immersed into an oil bath set at 48 °C to initiate the DMAC polymerization. After 18 h, the polymerization was quenched by exposing the reaction mixture to air while cooling to 20 °C. The final DMAC conversion was more than 99% as judged by ^1^H NMR spectroscopy (calculated by comparing the integrated vinyl signals assigned to the DMAC monomer at 5.6–6.7 ppm to the integrated polyacrylamide backbone signals at 1.1–2.7 ppm). Aqueous GPC analysis indicated an *M*_n_ of 74 kg mol^–1^ and an *M*_w_/*M*_n_ of 1.54 (see below for eluent and calibration details).

#### Preparation of Dilute Aqueous Dispersions for DLS and ζ Potential Studies

Aqueous dispersions of PMPC_139_–PDMAC_1000_, PATAC_195_–PDMAC_1000_, and PAMPS_250_–PDMAC_1000_ particles in 2.0 M ammonium sulfate were diluted to 0.1% w/w using 2.0 M ammonium sulfate, which had been adjusted to an apparent pH of 3.

#### Preparation of Titrant Solutions

A stock titrant solution was prepared gravimetrically comprising an aqueous solution of 1.0 M KOH (22.97 g), deionized water (99.98 g), and ammonium sulfate (30.40 g). Deionized water was filtered through a 0.2 μm polyethersulfone syringe filter prior to use.

### Characterization Methods

#### ^1^H NMR Spectroscopy

Spectra were recorded at 25 °C in D_2_O using a 400 MHz Bruker Avance-400 spectrometer with 64 scans being averaged per spectrum.

#### Gel Permeation Chromatography (GPC)

Molecular weights and dispersities were determined for the various homopolymers and diblock copolymers using an Agilent 1260 Infinity GPC instrument. The setup comprised a pump, a degasser, three columns in series (PL-Aquagel Mixed-H, OH-30, and OH-40), and a refractive index detector. The column and detector temperature was set at 30 °C, and the flow rate was 1.0 mL min^–1^. Calibration was achieved using nine near-monodisperse poly(ethylene oxide) standards (2.1–969 kDa), and data were analyzed using Agilent Technologies GPC/SEC software. For PMPC*_x_* and its copolymers, the eluent was an aqueous solution containing 0.20 M NaNO_3_ and 0.05 M Trizma buffer at pH 7.0. For PATAC*_x_* and its copolymers, the eluent was an aqueous solution containing 0.50 M CH_3_COOH and 0.30 M NaH_2_PO_4_ at pH 2.0. For PAMPS*_x_* and its copolymers, the eluent was a 70% v/v aqueous solution containing 0.20 M NaNO_3_ and 0.01 M NaH_2_PO_4_ at pH 7.0, with 30% v/v methanol.

#### Potentiometric Titration

An acidified aqueous dispersion (25.0 mL) was placed in a 250 mL glass beaker and stirred with a magnetic flea. Titrant solution was passed through a 0.2 μm polyethersulfone syringe filter into a volumetric 50 mL burette and a standard glass pH electrode was immersed within the aqueous dispersion. A total titrant of 7.0 mL was added in aliquots of no more than 0.5 mL, with smaller aliquots being used where necessary to accurately determine the equivalence point. The apparent pH of the aqueous dispersion was recorded after adding each aliquot, with pH equilibration being achieved within 30 s of each addition. All measurements were performed at 22 ± 1 °C. Approximately 0.25 mL of the aqueous dispersion was removed at suitable intervals for electrophoretic light scattering (ELS) and dynamic light scattering (DLS) analyses. No attempt was made to remove dissolved CO_2_ or to prevent its dissolution into the samples. It was assumed that the samples were near to or at the saturation level of dissolved CO_2_.

#### Dynamic Light Scattering (DLS)

Hydrodynamic diameters were determined using a Zetasizer Nano ZS (Malvern Panalytical, Malvern, U.K.). Samples were analyzed without further dilution, and three measurements were made in each case at a scattering angle of 173°. The instrument was configured to automatically determine the experimental duration and optical attenuation. The experimental correlation functions were analyzed using the cumulants method to yield the *z*-average hydrodynamic diameter (*D_z_*) and polydispersity index (PDI). The Stokes–Einstein equation was employed, which is valid for dilute, noninteracting, and monodisperse spheres. Method 1 involved using a quartz cuvette (10 mm path length; volume = 1.00 mL) and 0.05% w/w aqueous copolymer dispersions were analyzed at 20 °C. Method 2 involved using disposable polystyrene semi-micro cuvettes (4 mm path length; volume = 0.25 mL) and 0.1% w/w aqueous copolymer dispersions were analyzed at 25 °C.

#### Electrophoretic Light Scattering (ELS)

Electrophoretic mobilities were determined by electrophoretic light scattering (NG-ELS) using an instrument provided by Enlighten Scientific LLC (Hillsborough, NC). The functional design and operation of this instrument are similar to the original phase analysis light scattering (PALS) apparatus^44^ that employed a crossed-beam optical configuration in contrast to the more common reference beam configuration used for other ELS instruments. The electrode assembly used for the NG-ELS equipment is similar to that described by Uzgiris.^45^ Disposable polystyrene semi-micro cuvettes (4 mm path length; volume = 0.25 mL) were used as the sample holders. Two identical parallel plate platinized platinum^46^ electrodes, placed 4 mm apart, were used to provide the driving voltage. The sample temperature was determined using a miniature NTC-type thermistor placed in direct contact with an ≈0.1% w/w aqueous copolymer dispersion. This temperature sensor was positioned at the mid-point between the electrodes and approximately 1 mm above the intersection point of the two laser beams. Temperature control was achieved by placing the sample cuvette in an aluminum block that ensured efficient heat transfer with the (cooler) water circulating through channels within the block. The water temperature depended on the amount of Joule heating of the sample and hence on both the sample conductivity and the voltage applied across the electrodes. Complex impedance analysis of the electrode waveform was used to quantify electrode polarization and Joule heating. Mobility measurements were made using sinusoidal electrode signal waveforms with a nominal amplitude of 4.5 V at frequencies of 64 and 128 Hz. Small adjustments (up to ± 0.3 V) to the amplitude were made prior to data collection to ensure that the cell temperature remained at 25 ± 1 °C during each measurement. The scattered light was analyzed using both the PALS and the laser Doppler electrophoresis (LDE) methods simultaneously. Data were collected for 1 min, and the same data set was used to calculate the electrophoretic mobility by each method. For each sample, five independent measurements were made at each electrode signal frequency, yielding a total of ten measurements per sample from which a mean value was calculated.

#### Small-Angle X-Ray Scattering (SAXS)

SAXS patterns were recorded for 1.0% w/w aqueous copolymer dispersions at Diamond Light Source (station I22, Didcot, U.K.) using a monochromatic X-ray beam (λ = 0.124 nm), a two-dimensional (2D) Pilatus 2M pixel detector (Dectris, Switzerland), and a *q* range of 0.02–2.00 nm^–1^, where *q* = (4π sin θ)/λ corresponds to the modulus of the scattering vector, and θ is half of the scattering angle. SAXS data were reduced (integrated, normalized, and background-subtracted) using Dawn software supplied by Diamond Light Source. The X-ray scattering intensity for water was used for absolute scale calibration of the scattering patterns with data manipulation via SAXS Utilities software. Irena SAS macros for Igor Pro were utilized for modeling.

#### Optical Microscopy (OM)

Images were recorded at ×400 magnification using a Cole-Parmer bifocal compound microscope equipped with a Moticam-BTW digital camera.

#### UV Absorption Spectroscopy

Spectra were recorded between 200 and 400 nm using a PC-controlled UV-1800 spectrophotometer at 25 °C using a 1 cm path length quartz cell. A Beer–Lambert curve was constructed using a series of five PETTC solutions in methanol. The absorption maximum at 306 nm assigned to the trithiocarbonate group was used for this calibration plot, and PETTC concentrations were selected such that the absorbance remained below 1.1. The molar extinction coefficient for PETTC was determined to be 10,900 ± 100 mol^–1^ dm^3^ cm^–1^; hence the mean DP for the PMPC homopolymer was calculated to be 139 ± 1.

#### Rheology

An MCR 502 rheometer (Anton Paar, Gratz, Austria) equipped with a Couette geometry was used for rotational rheology experiments. Measurements were performed at 20 °C and shear sweeps were conducted from 0.05 to 500 s^–1^ using approximately 10 mL of either 20% w/w aqueous copolymer dispersions or 10% w/w aqueous copolymer solutions.

## Results and Discussion

**Scheme 2:**
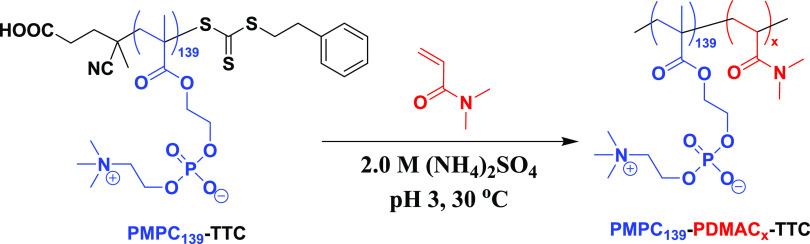
Reaction Scheme for the Synthesis of PMPC_139_–PDMAC*_x_* (*x* = 500 to 7000) Diblock Copolymer Particles via RAFT Aqueous Dispersion Polymerization of DMAC at 30 °C in the Presence of 2.0 M Ammonium Sulfate Conditions: targeting 20% w/w solids using a PMPC_139_–TTC/KPS molar ratio of 4.0 and a [KPS]/[AsAc] molar ratio of 1.0.

The RAFT solution polymerization of MPC was conducted in methanol at 64 °C using a trithiocarbonate-based RAFT agent (PETTC). The mean degree of polymerization (DP) of the PMPC homopolymer was determined via end-group analysis using UV spectroscopy to be 139 ± 1. Aqueous GPC analysis indicated a relatively narrow molecular weight distribution (*M*_w_/*M*_n_ = 1.18) for the precursor. However, the poly(ethylene oxide) calibration standards used for GPC analysis meant that only relative *M*_n_ values could be obtained. The RAFT aqueous dispersion polymerization of DMAC was conducted in the presence of 2.0 M ammonium sulfate at 30 °C using the PMPC_139_ precursor as a salt-tolerant steric stabilizer block, as outlined in Scheme 2.

PMPC was chosen as the steric stabilizer block because its zwitterionic structure is known to confer aqueous solubility even at 5.0 M NaCl.^35^ PDMAC was chosen as the core-forming block because it is a non-ionic water-soluble polymer that becomes water-insoluble in the presence of added salt.^47^ Moreover, the resulting PMPC–PDMAC diblock copolymer chains were anticipated to be amenable to aqueous GPC analysis.^48^ Inspecting the data presented in Table 1, using 2.0 M ammonium sulfate should be sufficient to produce an aqueous dispersion polymerization formulation since the PMPC precursor and the DMAC monomer are soluble in the aqueous continuous phase and the growing PDMAC chains should become insoluble. Accordingly, the series of aqueous PISA syntheses shown in Table 2 were conducted at 30 **°**C using a low-temperature persulfate/ascorbic acid (KPS/AsAc) redox initiator while targeting 20% w/w solids at pH 3.

**Table 1:** Aqueous Solubility of MPC Monomer, DMAC Monomer, PMPC_139_ Homopolymer, and PDMAC_500_ Homopolymer at 5.0% w/w Solids in the Presence of 0–4.0 M Ammonium Sulfate as Judged by Visual Inspection at pH 7 and 25 °C

	aqueous (NH_4_)_2_SO_4_ concentration/mol dm^–3^				
	0	1.0	2.0	3.0	4.0
MPC monomer	soluble	soluble	soluble	soluble	soluble
PMPC_139_	soluble	soluble	soluble	soluble	soluble
DMAC monomer	soluble	soluble	soluble	soluble	insoluble
PDMAC_500_	soluble	soluble	insoluble	insoluble	insoluble

The PMPC_139_ precursor afforded colloidally stable dispersions of increasing turbidity when targeting PDMAC DPs ranging from 500 to 6000. However, precipitation was observed when targeting PDMAC DPs above 6000 or when the target copolymer concentration was increased to 25% w/w solids. The PDMAC core block DPs were determined relative to that of the stabilizer block by end-group analysis using ^1^H NMR spectroscopy. Reasonably good agreement (within experimental error) with the target PDMAC DPs was confirmed by comparing the integrated methine proton signal on the PDMAC backbone at 2.2–2.7 ppm and the PMPC_139_ azamethylene signal at 3.6 ppm. Comparing these NMR-derived *M*_n_ values to those determined by GPC analysis suggests a significant systematic error for the latter technique. This is understandable because poly(ethylene oxide) calibration standards are unlikely to be accurate for the analysis of PDMAC-rich diblock copolymers. Macroscopic precipitation was observed for the attempted synthesis conducted at 30% w/w solids. Essentially full DMAC conversion (>98%) was obtained for each of these syntheses as judged by ^1^H NMR spectroscopy studies. Targeting PDMAC DPs ≤1000 produced translucent gels, but lowering the solid concentration led to free-flowing dispersions. These gels are most likely caused by these relatively short PDMAC chains not being fully desolvated in the presence of 2.0 M ammonium sulfate.

There is a systematic increase in particle diameter when targeting higher PDMAC DPs. Similar observations have been reported for various other PISA formulations that produce kinetically-trapped spheres.^33,49,50^ Moreover, there is also good evidence that targeting larger particles (i.e., higher PDMAC DPs) leads to a progressive broadening of the particle size distribution.

**Figure 1:**
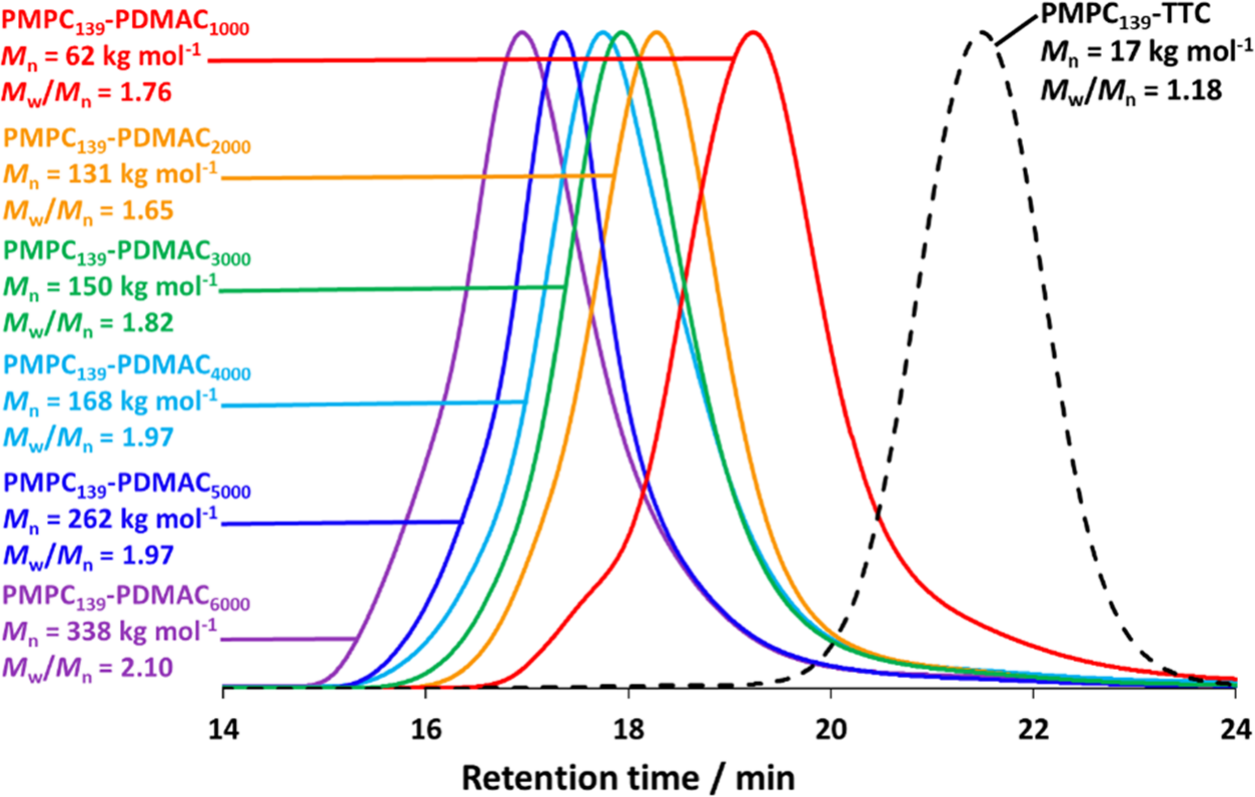
Aqueous GPC curves recorded for the PMPC_139_ precursor and a series of PMPC_139_–PDMAC*_x_* diblock copolymers prepared by chain extension via RAFT aqueous dispersion polymerization of DMAC at 30 °C in the presence of 2.0 M ammonium sulfate. *M*_n_ values are calculated relative to a series of near-monodisperse poly(ethylene oxide) calibration standards (see Figure S2 for the corresponding normalized GPC curves).

Aqueous GPC data obtained for the series of PMPC_139_–PDMAC*_x_* diblock copolymers shown in Table 2 are summarized in Figure 1. Unimodal molecular weight distributions are obtained in each case, with a systematic shift to higher *M*_n_ observed when targeting higher PDMAC DPs, which can also be observed in the normalized GPC traces (see Figure S2). However, dispersities are above 1.50, which indicates imperfect RAFT control. We have reported similar dispersities when targeting relatively high DPs for the core-forming block in other PISA formulations.^51−54^ Because *M*_n_ values are calculated relative to a series of near-monodisperse poly(ethylene oxide) calibration standards, a significant systematic error is expected in this case. Indeed, the theoretical *M*_n_ for PMPC_139_–PDMAC_6000_ is 636 kg mol^–1^, whereas the corresponding experimental GPC value is 338 kg mol^–1^.

**Table 2:** Summary of Conversion, GPC, and DLS Data Obtained for the RAFT Aqueous Dispersion Polymerization of DMAC at 30 °C Using a PMPC_139_ Precursor at 20–30% w/w Solids

			calculated for PDMAC block:						
solids/w/w%	PDMAC DP (*x*)	conversion^a^/%	DP^a^	*M*_n_^a^/kg mol^–1^	GPC *M*_n_^b^/kg mol^–1^	*M*_w_/*M*_n_^b^	*D*_z_^c^/nm	PDI^c^	physical appearance
20	500	>99	520	52	31	1.96	70	0.09	translucent gel
	1000	>99	1000	99	62	1.76	98	0.12	translucent gel
	2000	>99	1980	196	131	1.65	240	0.10	free-flowing & turbid
	3000	>99	3060	303	150	1.82	253	0.17	free-flowing & turbid
	4000	>99	4020	398	168	1.97	350	0.27	free-flowing & turbid
	5000	>99	4910	487	262	1.97	560	0.31	free-flowing & turbid
	6000	>99	6090	604	338	2.10	680	0.40	free-flowing & turbid
	7000	>98	6630	657	unstable dispersion				
25	5000	>99	5110	506	unstable dispersion				
30	5000	macroscopic precipitation							

a Determined by ^1^H NMR spectroscopy (comparison between the integrated vinyl signals assigned to DMAC monomer at 5.6–6.7 ppm, the integrated PDMAC methine proton signal at 2.2–2.7 ppm, and the PMPC_139_ azamethylene signal at 3.6 ppm).

b Determined by aqueous GPC using a series of near-monodisperse poly(ethylene oxide) calibration standards.

c *D*_z_ denotes *z*-average diameter and PDI denotes polydispersity index as determined by DLS according to method 1 (see main text).

**Figure 2:**
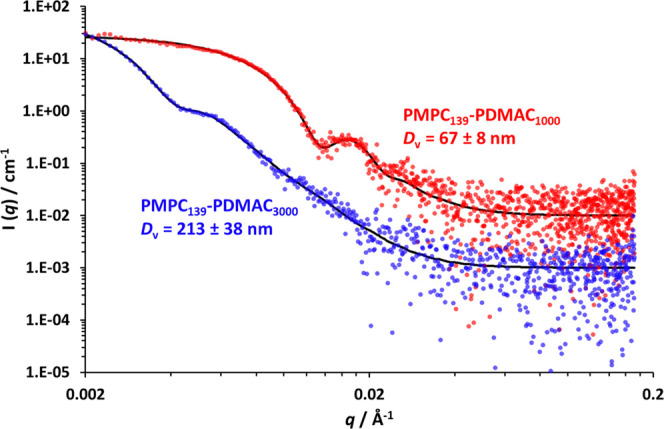
SAXS patterns recorded for 1.0% w/w aqueous dispersions of PMPC_139_–PDMAC*_x_* particles (where *x* = 1000 or 3000) at 25 °C. The black solid lines denote the data fit obtained using a well-known spherical micelle model.^55−57^*D*_v_ denotes the volume-average diameter. The red pattern has been scaled by a factor of ten relative to the blue pattern for the sake of clarity.

Small-angle X-ray scattering (SAXS) was used to characterize selected PMPC_139_–PDMAC*_x_* particles (where *x* = 1000 or 3000), see Figure 2. A well-known spherical micelle model^55−57^ was used to provide a satisfactory data fit to an *I*(*q*) vs *q* plot. This approach indicated a volume-average diameter (*D*_v_) of 67 ± 8 nm for the PMPC_139_–PDMAC_1000_ particles and 213 ± 38 nm for the PMPC_139_–PDMAC_3000_ particles. As expected, these diameters are somewhat lower than the corresponding z-average diameters reported by DLS (see Table 2).^58^

**Figure 3:**
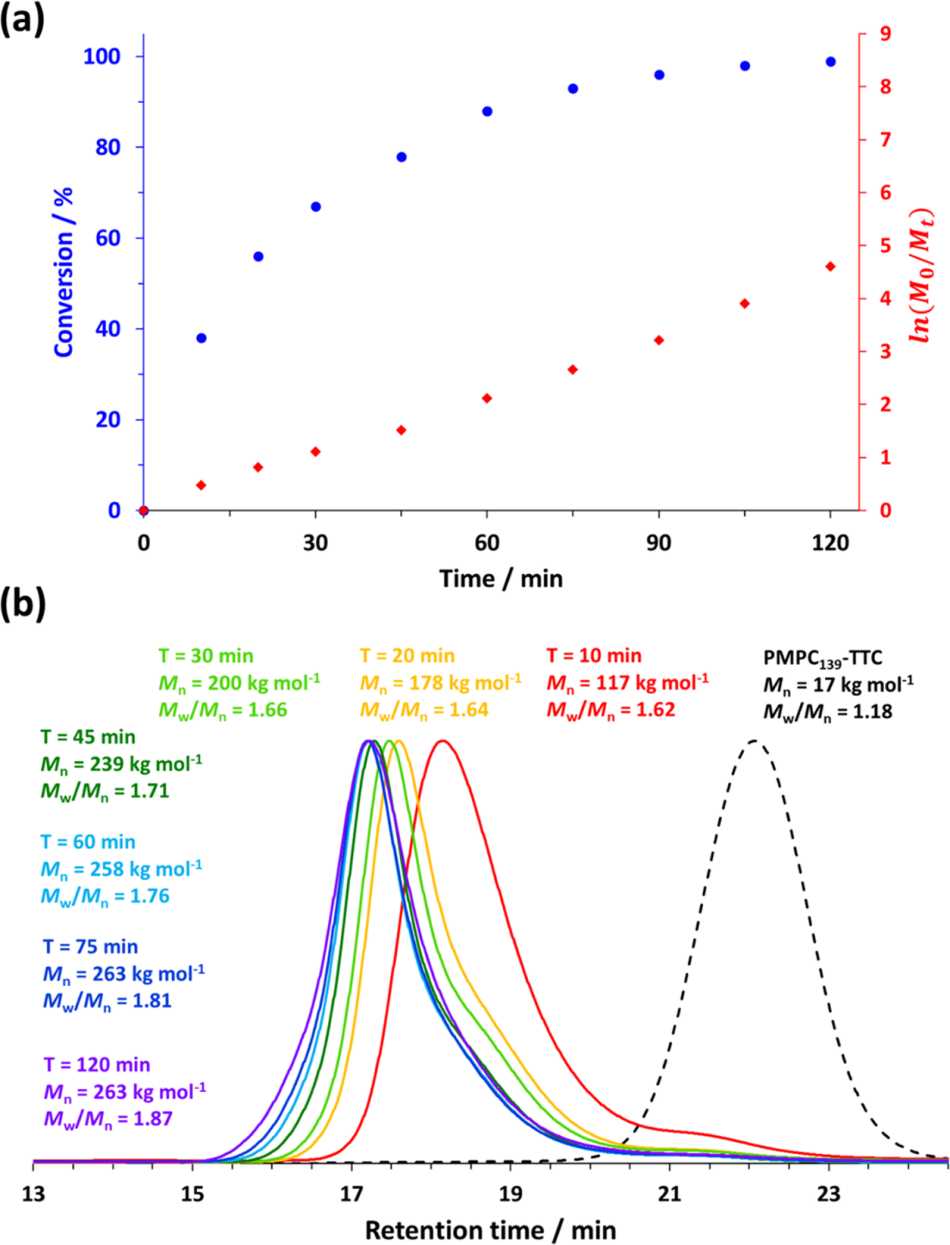
(a) Conversion vs time curve and corresponding semilogarithmic plot determined by ^1^H NMR spectroscopy for the RAFT aqueous dispersion polymerization of DMAC at 30 °C in 2.0 M ammonium sulfate when targeting a PDMAC DP of 5000 at 20% w/w solids. (b) Aqueous GPC curves obtained by periodic sampling of the reaction mixture to monitor the evolution in the molecular weight distribution (see Figure S3 for the corresponding normalized GPC curves).

^1^H NMR spectroscopy was used to study the kinetics of the DMAC polymerization at 30 °C when targeting PMPC_139_–PDMAC_5000_ particles at 20% w/w solids (see Figure 3a). Periodic sampling of the reaction mixture confirmed that a DMAC conversion of 98% was achieved within 2 h under such conditions, while the corresponding linear semilogarithmic plot indicated first-order kinetics with respect to monomer. Each aliquot taken from the reaction mixture was also subjected to analysis by aqueous GPC (see Figure 3b). Each GPC curve was unimodal, and there was a clear shift in the entire molecular weight distribution toward higher molecular weight, indicating a reasonably high blocking efficiency for the PMPC_139_ precursor and hence well-defined diblock copolymer chains. This is perhaps more apparent for the normalized GPC curves (see Figure S3). However, dispersities increased monotonically with monomer conversion and always remained above 1.50.

**Figure 4:**
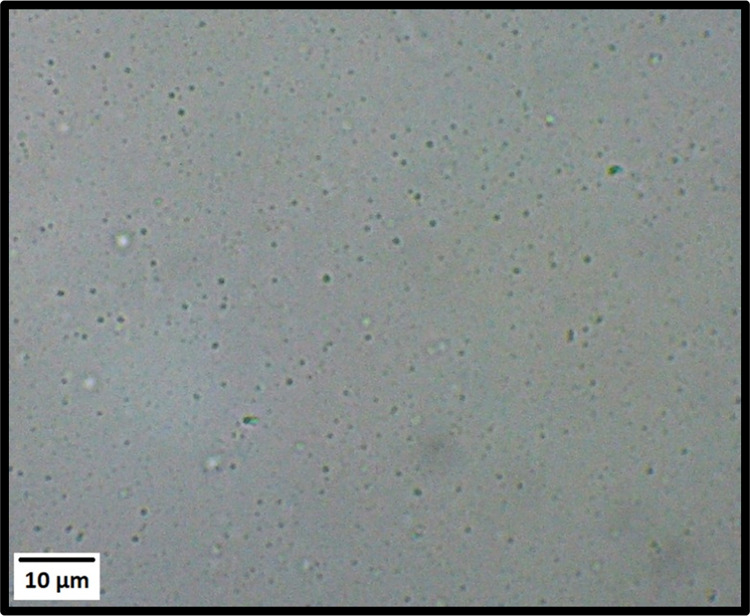
Optical microscopy image recorded for PMPC_139_–PDMAC_5000_ particles prepared at 20% w/w solids by RAFT aqueous dispersion polymerization of DMAC at 30 °C in the presence of 2.0 M ammonium sulfate.

In principle, transmission electron microscopy (TEM) can be used to assign the morphology of diblock copolymer particles prepared via PISA. In practice, the particles prepared herein are unstable with respect to dilution with deionized water (see below). On the other hand, dilution using an aqueous solution of 2.0 M ammonium sulfate is also problematic because this leads to extensive salt crystal formation during TEM grid preparation. In view of these technical problems, we examined the PMPC_139_–PDMAC_5000_ particles by optical microscopy, see Figure 4. This technique indicates the presence of a population of micron-sized particles, but it is insensitive to the submicron-sized particle populations indicated by DLS and SAXS studies.

**Figure 5:**
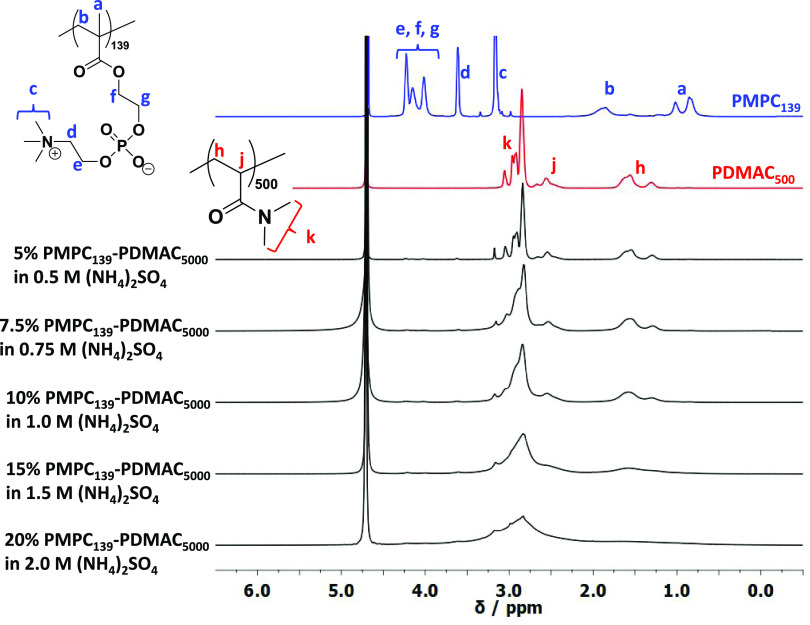
^1^H NMR spectra recorded for a PDMAC_500_ (red spectrum) and a PMPC_139_ (blue spectrum) homopolymers in the absence of salt, as well as a PMPC_139_–PDMAC_5000_ diblock copolymer prepared at 20% w/w in D_2_O in the presence of 2.0 M ammonium sulfate, see the lowest black spectrum. As the 20% w/w PMPC_139_–PDMAC_5000_ dispersion is diluted with further D_2_O, both the background salt concentration and the copolymer concentration are systematically reduced (see four other black spectra). Just a two-fold dilution of the turbid dispersion is sufficient to cause molecular dissolution of the particles as the PDMAC block becomes solvated in 1.0 M ammonium sulfate. A further two-fold dilution of this transparent solution with D_2_O results in PMPC_139_–PDMAC_5000_ chains dissolved in 0.5 M ammonium sulfate, for which the PDMAC signals are now indistinguishable from those of PDMAC_500_ homopolymer in water (compare the uppermost black spectrum with the red spectrum).

^1^H NMR spectroscopy was employed to investigate the extent of solvation of the core-forming PDMAC block before and after particle dissolution on dilution of a 20% w/w aqueous dispersion with deionized water. Accordingly, PMPC_139_–PDMAC_5000_ particles were prepared in D_2_O in the presence of 2.0 M ammonium sulfate using the same reaction conditions outlined in Scheme 2. ^1^H NMR spectra were recorded for the initial aqueous dispersion and the resulting aqueous solutions after up to a four-fold dilution using D_2_O (see Figure 5). The lower five spectra were normalized to the signal assigned to the two azamethylene protons (−C*H*_2_N(CH_3_)_3_) adjacent to the quaternary amine within the PMPC block, which remains fully solvated at all salt concentrations. The uppermost spectrum was recorded for a PDMAC_500_ homopolymer in D_2_O; the signals marked *a* and *b* correspond to the methylene and methine backbone protons, and *c* corresponds to the two equivalent pendent methyl groups.

Clearly, signal a is almost completely attenuated in the presence of 2.0 M ammonium sulfate. However, this signal becomes much more prominent as the ammonium sulfate concentration is lowered, indicating a much higher degree of hydration for the PDMAC block on dilution. Similar observations were made for signals *b* and *c*. However, the former signal overlaps with other signals, while the latter is only partially suppressed in the presence of 2.0 M ammonium sulfate. The spectra recorded using 0.5 M ammonium sulfate and the pure PDMAC homopolymer were almost identical, which suggests that this polymer is essentially fully solvated at this salt concentration. This indicates that lowering the ammonium sulfate concentration from 2.0 to 0.5 M via four-fold dilution of the as-synthesized 20% w/w aqueous dispersions of PMPC_139_–PDMAC_5000_ particles using deionized water should be sufficient to cause complete particle dissolution.^47,54^

**Figure 6:**
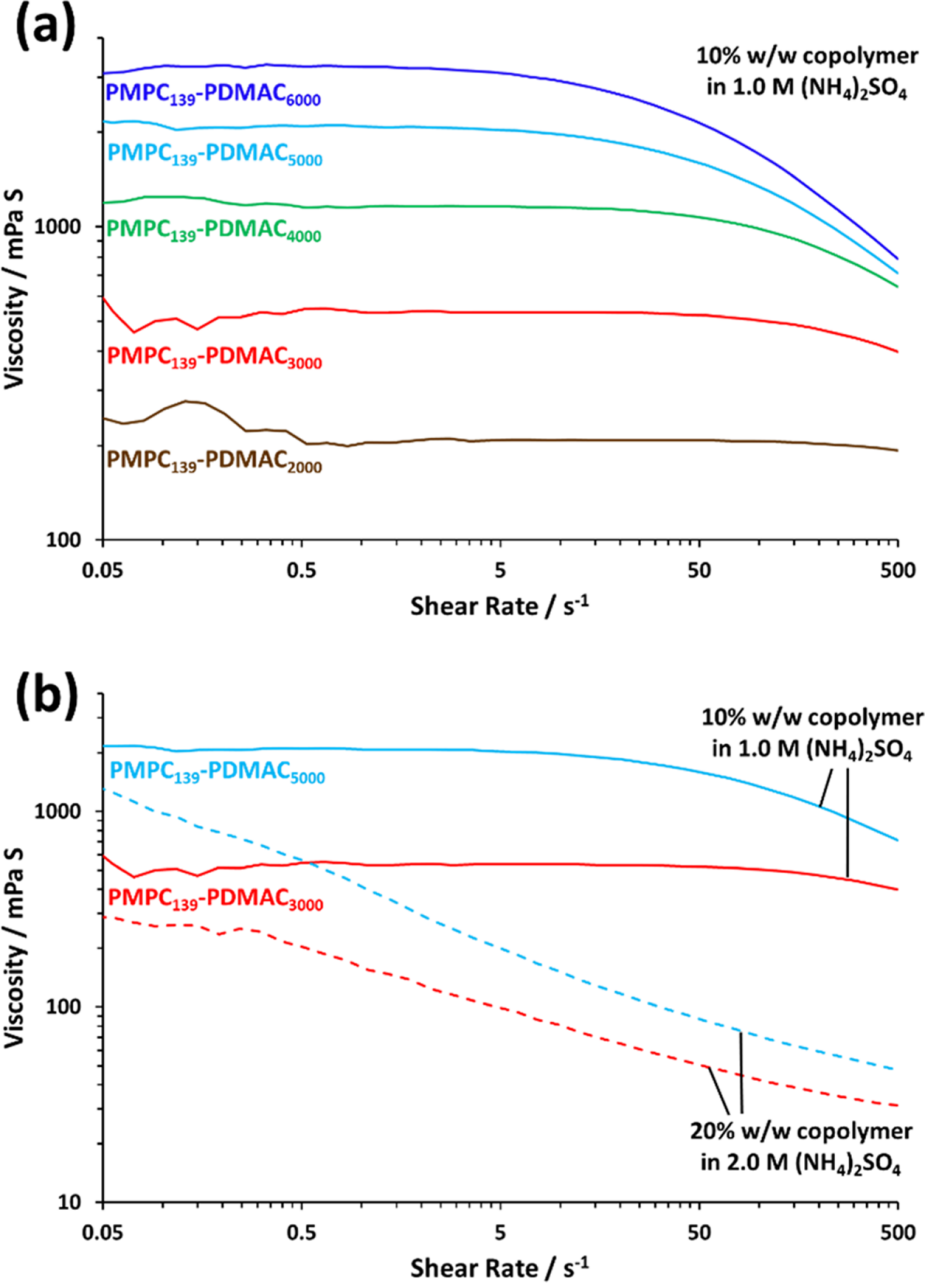
(a) Viscosity vs shear rate data obtained by rotational rheology studies of 10% w/w aqueous solutions of molecularly-dissolved PMPC_139_–PDMAC*_x_* chains in the presence of 1.0 M ammonium sulfate. (b) Viscosity vs shear rate data obtained by rotational rheology studies of 20% w/w aqueous dispersions of either PMPC_139_–PDMAC_3000_ or PMPC_139_–PDMAC_5000_ particles in 2.0 M ammonium sulfate compared to that for 10% w/w aqueous solutions of the same two copolymers in the presence of 1.0 M ammonium sulfate.

Rotational rheology experiments were conducted on samples using shear sweeps from 0.05 to 500 s^–1^ at 20 °C. The viscosities of a range of 10% w/w aqueous solutions comprising molecularly-dissolved PMPC_139_–PDMAC*_x_* chains in the presence of 1.0 M ammonium sulfate obtained after two-fold dilution of the as-synthesized dispersions using deionized water are shown in Figure 6a. A monotonic increase in solution viscosity is observed at all shear rates when increasing the PDMAC DP for the molecularly-dissolved chains. The viscosity of the aqueous solution remains relatively constant for shear rates ranging from 0.05 to 5.0 s^–1^, with shear-thinning behavior being observed at higher shear rates. Figure 6b compares the viscosities of as-synthesized 20% w/w aqueous dispersions of PMPC_139_–PDMAC*_x_* particles (where *x* = 3000 or 5000) in 2.0 M ammonium sulfate with the corresponding two 10% w/w aqueous copolymer solutions in 1.0 M ammonium sulfate. Clearly, the viscosity of each dispersion is significantly lower than that of the more dilute solution at all shear rates. Moreover, the two dispersions are much more strongly shear-thinning at higher shear, leading to an order of magnitude reduction in viscosity at 5 s^–1^. Similar behavior has been reported in the literature when comparing colloidal particles with the corresponding solvated copolymer chains.^60,61^

**Scheme 3:**
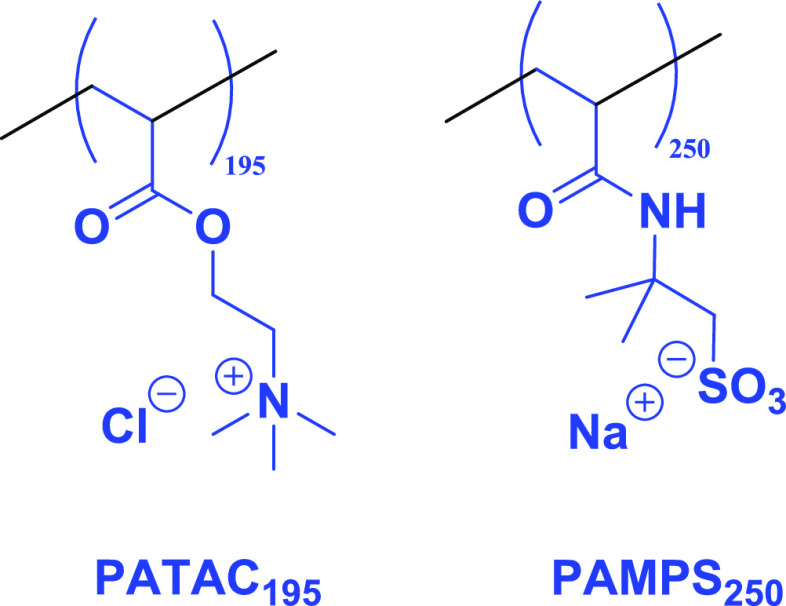
Chemical Structures of the Cationic PATAC_195_ and Anionic PAMPS_250_ Precursors Used to Stabilize PDMAC-Rich Diblock Copolymer Particles Prepared via RAFT Aqueous Dispersion Polymerization of DMAC in 2.0 M Ammonium Sulfate

Two alternative steric stabilizers were also evaluated for the RAFT aqueous dispersion polymerization of DMAC conducted in the presence of 2.0 M ammonium sulfate. To complement the zwitterionic nature of the salt-tolerant PMPC_139_ steric stabilizer, we evaluated a cationic polyelectrolyte (PATAC_195_) and an anionic polyelectrolyte (PAMPS_250_), see chemical structures shown in Scheme 3. Both these polyelectrolytes have been reported to exhibit salt-tolerant behavior.^34,62−64^ A PDMAC DP of 1000 was targeted, and ^1^H NMR spectroscopy studies of the final reaction mixtures confirmed that more than 99% DMAC conversion was obtained in each case.

It is common practice to estimate the ζ potential of colloidal particles in aqueous solution as a function of pH. However, the correct interpretation of the experimental data for sterically-stabilized particles dispersed in highly salty aqueous media can be problematic for two reasons. First, the relatively high ionic strength reduces the hydrogen ion activity, which affects the accuracy of the glass Ag/AgCl reference electrode typically used to measure the pH. Moreover, additional errors may be incurred owing to a change in the junction potential of such electrodes when in contact with such salty media. Second, the steric stabilizer chains at the particle–liquid interface provide a permeable medium through which the solution phase can flow. If electrical charge arises from the ionic groups within such steric stabilizer chains, the electrokinetic models commonly used to calculate ζ potential from electrophoretic mobility become invalid.^65^ To overcome these technical problems, we used a state-of-the-art instrument to determine apparent ζ potentials for the three types of PDMAC-rich particles prepared in high salt using the PMPC_139_, PATAC_195_, or PAMPS_250_ precursor in turn (see Supporting Information for further information).

In the present study, the electrophoretic mobility of the particles was measured as a function of the addition of varying amounts of KOH. The apparent pH was determined using a glass Ag/AgCl reference electrode without any compensation to offset the effect of the high ionic strength on the electrode response (although a temperature sensor within the electrode assembly did enable temperature compensation). Accordingly, ζ potentials calculated using the Smoluchowski model^66^ are regarded as apparent zeta potentials. The hydrodynamic *z*-average diameter of the particles was also determined in the presence of 2.0 M ammonium sulfate as a function of pH during these measurements.

**Figure 7:**
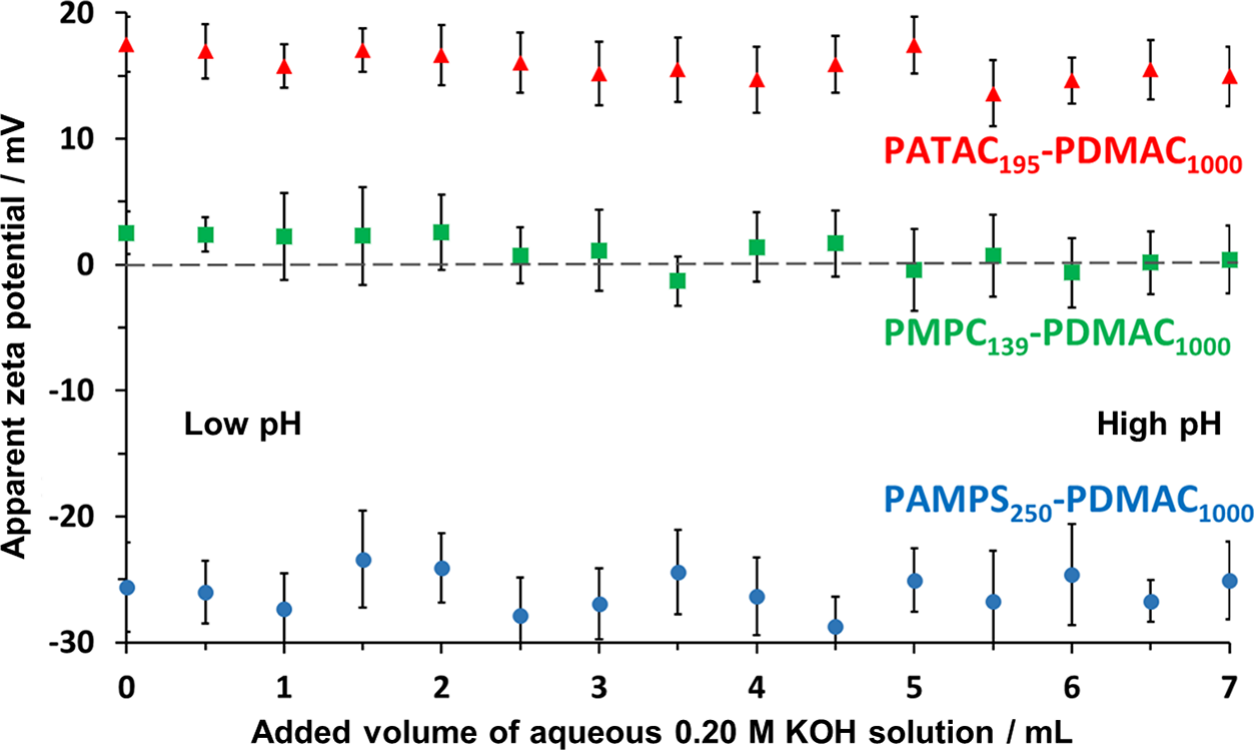
Apparent ζ potentials observed on addition of varying volumes of aqueous 0.2 M KOH solution in the presence of 2.0 M ammonium sulfate for 0.1% w/w aqueous dispersions of PATAC_195_–PDMAC_1000_ (red triangles), PMPC_139_–PDMAC_1000_ (green squares), or PAMPS_250_–PDMAC_1000_ (blue circles) particles.

The apparent ζ potentials determined by electrophoretic light scattering for each of these three dispersions as a function of added KOH is shown in Figure 7. As expected, the electrophoretic footprint for each type of particle is dictated by the chemical nature of the steric stabilizer chains. Thus the cationic PATAC_195_–PDMAC_1000_ particles exhibit positive apparent ζ potentials of +15.8 ± 1.1 mV, whereas the anionic PAMPS_250_–PDMAC_1000_ particles exhibit negative apparent ζ potentials of −25.9 ± 1.5 mV. Finally, the zwitterionic PMPC_250_–PDMAC_1000_ particles exhibit apparent ζ potentials close to zero (+1.1 ± 1.2 mV). Similar observations have been reported for other PMPC-stabilized nano-objects at low salt.^67,68^

If these particles were hard spheres, the conventional interpretation for such electrophoretic observations is that the PAMPS_250_–PDMAC_1000_ particles possess a sufficiently high anionic ζ potential to prevent aggregation, the PATAC_195_–PDMAC_1000_ particles possess a moderate cationic ζ potential that is likely to retard but not prevent aggregation, while the PMPC_250_–PDMAC_1000_ particles are essentially uncharged and hence likely to be colloidally unstable. However, this is a naïve and incorrect interpretation, not least because the salt-tolerant PAMPS_250_, PATAC_195_, and PMPC_139_ chains confer additional steric stabilization.^69^

## Conclusions

We report the synthesis of a series of sterically-stabilized diblock copolymer particles via RAFT aqueous dispersion polymerization of DMAC in highly salty media. This is achieved by selecting a suitable salt-tolerant water-soluble polymer to act as an effective steric stabilizer. Such stabilizers can possess zwitterionic (e.g., PMPC), cationic (e.g., PATAC), or anionic (e.g., PAMPS) character, which leads to the corresponding diblock copolymer particles exhibiting essentially zero, negative, or positive apparent ζ potentials, respectively. It is non-trivial to make such aqueous electrophoresis measurements in highly salty media. Indeed, such experiments require state-of-the-art instrumentation. Relatively high DPs can be targeted for the salt-insoluble block to ensure that this component dominates the formulation. This approach enables high molecular weight water-soluble polymers to be prepared in a highly convenient low-viscosity form. Subsequent dilution using deionized water lowers the background salt concentration and causes *in situ* molecular dissolution of the particles, which leads to a substantial thickening effect and the formation of highly viscous transparent aqueous solutions. In principle, such aqueous PISA formulations are highly attractive: there are various potential commercial applications for high molecular weight water-soluble polymers while the well-known negative aspects of using RAFT agents (i.e., their cost, color, and malodor) are minimized. For example, the organosulfur content of the dry PMPC_139_–PDMAC_6000_ diblock copolymer targeted herein is only ≈0.03%, which corresponds to just ≈ 63 ppm for a 20% w/w aqueous copolymer dispersion.

## Supporting Information Available

UV absorption spectra and calibration plot; dynamic viscosities of various ammonium sulfate solutions; normalized GPC curves obtained for both final copolymers and during kinetic experiments; and further technical details regarding the determination of ζ potentials in highly salty media (PDF)
