# Supplementary material for: Synthesis of High Molecular Weight Water-Soluble Polymers as Low-Viscosity Latex Particles by RAFT Aqueous Dispersion Polymerization in Highly Salty Media
Source: Macromolecules. 2022 Aug 28;55(17):7380–91. doi: 10.1021/acs.macromol.2c01071 (PMC9476848; doi:10.1021/acs.macromol.2c01071)
Supplement: Supplementary file 1 — ma2c01071_si_001.pdf [file ma2c01071_si_001.pdf]

Supporting Information for:

Synthesis of High Molecular Weight Water-Soluble Polymers as Low-Viscosity Latex Particles by RAFT Aqueous Dispersion Polymerization in Highly Salty Media

Rory J. McBride,[a] John F. Miller,[b] Adam Blanazs,[c] Hans-Joachim Hähnle[c] and Steven P. Armes[a,*]

a. Chemistry Department, University of Sheffield, Brook Hill, Sheffield, South Yorkshire, S3 7HF, UK.

b. Enlighten Scientific LLC, Hillsborough, North Carolina 27278, USA.

c. BASF SE, RAM/OB - B001, Carl-Bosch-Strasse 38, 67056 Ludwigshafen am Rhein, Germany.

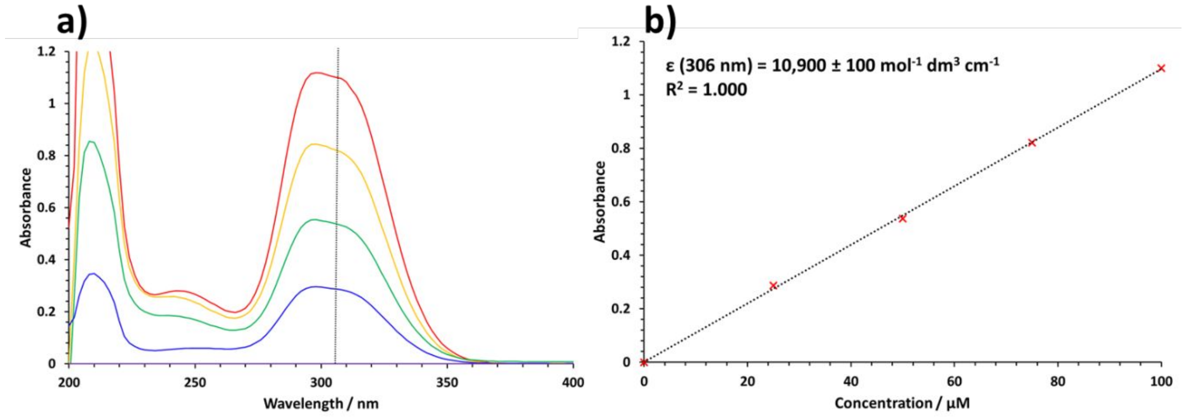

Figure S1. (a) UV absorption spectra recorded for 4-cyano-4-(2-phenylethanesulfanylthio-carbonyl)sulfanylpentanoic acid (PETTC) in methanol for a series of concentrations ranging from 0 µM to 100 µM. (b) Beer-Lambert calibration plot constructed for PETTC in methanol to calculate its molar extinction coefficient (ε) at the absorption maximum of 306 nm.

$(NH_4)_2SO_4$ concentration / M	Dynamic viscosity / Pa s	Refractive index
0.5	1.095	1.344
1.0	1.225	1.353
2.0	1.510	1.370
3.0	2.030	1.384

Table S1. Summary of dynamic viscosities and refractive indices for various aqueous $(NH_4)_2SO_4$ solutions at 20 °C.[1] These data were used for DLS analysis.

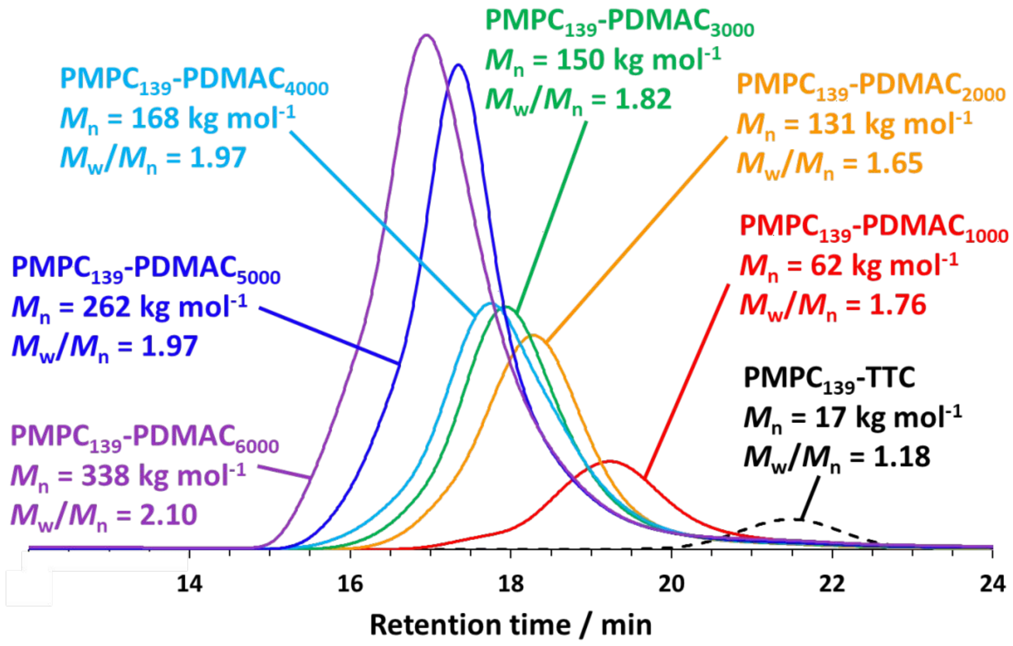

Figure S2. Normalized aqueous GPC curves (see Figure 1) recorded for the $PMPC_{139}$ precursor and a series of $PMPC_{139}$-$PDMAC_x$ diblock copolymers prepared by chain extension via RAFT aqueous dispersion polymerization of DMAC at 30 °C in the presence of 2.0 M ammonium sulfate. M_n values are calculated relative to a series of near-monodisperse poly(ethylene oxide) calibration standards. (see **Figure 1** in the main manuscript).

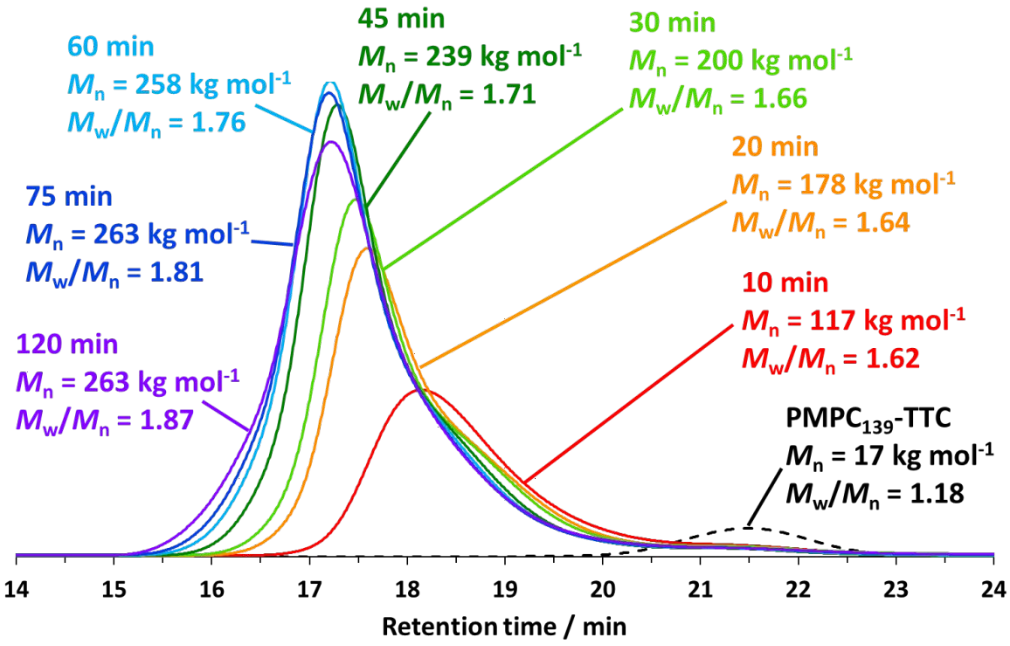

Figure S3. Normalized aqueous GPC curves obtained by periodic sampling of the reaction mixture to monitor the evolution in the molecular weight distribution (see **Figure 3b** in the main manuscript).

Further Technical Details Regarding Zeta Potential Measurements at High Salt Concentrations

The hydrogen ion activity can be determined by potentiometric acid-base titration using a suitable electrode and standardized aqueous solutions of HCl and KOH in the presence of the relevant electrolyte. Since the hydrogen ion concentration can be determined volumetrically, its apparent value indicated by the reference electrode can be corrected. However, this approach fails if there is any buffering action due to the presence of electrolyte, which is unfortunately the case for the dispersions investigated herein. In principle, the electrophoretic mobility can be determined at constant pH over a wide range of electrolyte concentration in order to select an appropriate electrokinetic model.[2] However, for the particles described herein this is not possible, because a relatively high ionic strength (e.g. 2.0 M ammonium sulfate) is required to ensure that the core-forming PDMAC chains remain sufficiently dehydrated to maintain particle stability. In this context, it is noteworthy that the hydrodynamic diameters determined by DLS (using method 2, see Experimental) for each dispersion after addition of various volumes of aqueous 0.2 M KOH solution remains relatively constant (see Figure S4).

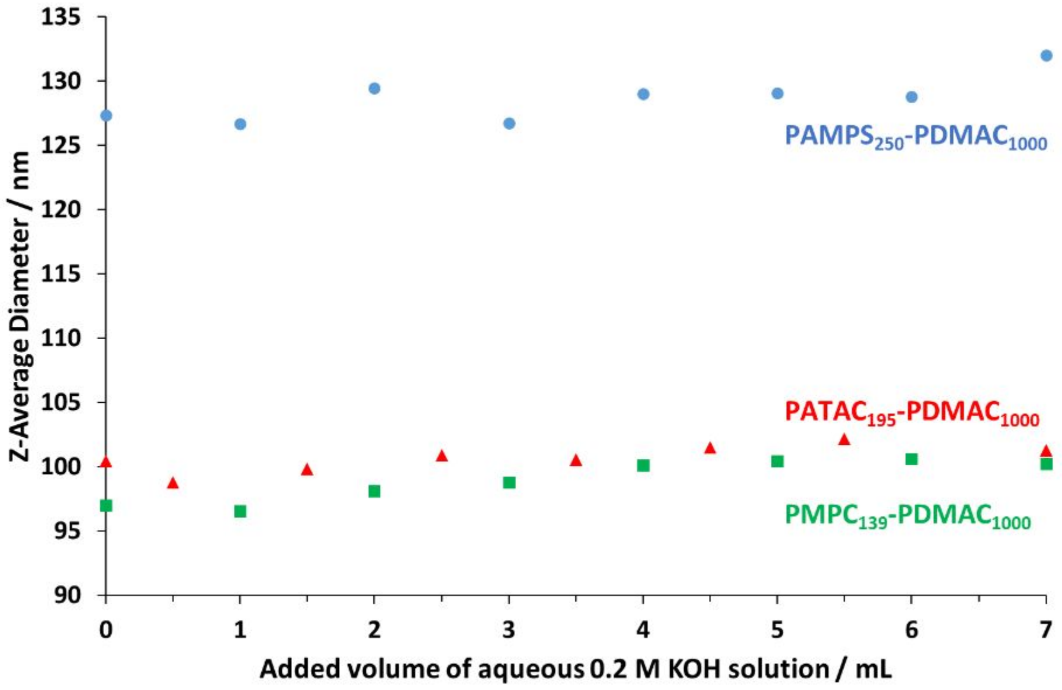

Figure S4. DLS z-average diameter determined for three types of PDMAC-core latex particles using method 2 (see Experimental) after dilution to 0.1% w/w using 2.0 M ammonium sulfate and adjustment of the solution pH to an apparent value of pH 3. This was followed by addition of varying volumes of aqueous 0.2 M KOH. Red triangles, green squares and blue circles denote PATAC$_{195}$-PDMAC$_{1000}$, PMPC$_{139}$-PDMAC$_{1000}$ and PAMPS$_{250}$-PDMAC$_{1000}$, respectively.

In order to enable conversion between apparent pH and calculated pH, potentiometric titration curves were obtained for dilute 0.1% w/w aqueous dispersions comprising PMPC$_{139}$-PDMAC$_{1000}$, PATAC$_{195}$-PDMAC$_{1000}$ and PAMPS$_{250}$-PDMAC$_{1000}$ particles (see Figure S5).

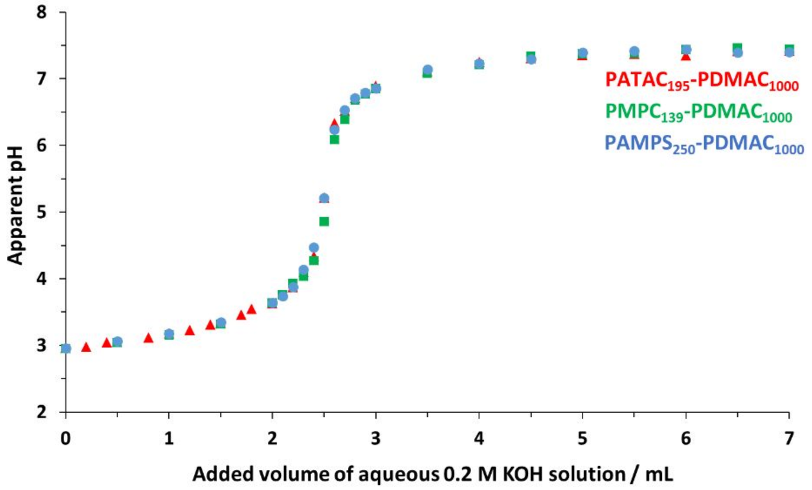

Figure S5. Apparent pH recorded for various aqueous dispersions of PDMAC-core latex particles in 2.0 M ammonium sulfate on addition of varying volumes of aqueous 0.2 M KOH. Application of a numerical curve-fitting protocol to these data yielded an approximate equivalence point at an added volume of 0.20 M KOH solution of 2.48 mL. Thus the initial HCl concentration for each dispersion is estimated to be 0.0198 M, which corresponds to a pH of 1.70 under ideal conditions (i.e., a hydrogen activity coefficient of unity). Red triangles, green squares and blue circles denote PATAC$_{195}$-PDMAC$_{1000}$, PMPC$_{139}$-PDMAC$_{1000}$ and PAMPS$_{250}$-PDMAC$_{1000}$, respectively.

A calculated pH value of 1.70 can be compared with the apparent pH of approximately 2.96 determined in the presence of 2.0 M ammonium sulfate. This discrepancy serves to illustrate the influence of such high ionic strength on pH measurements using glass Ag/AgCl reference electrodes.

Moreover, the relatively high ionic strength must also be considered when interpreting the electrophoretic mobility (and corresponding apparent zeta potential). Using classical electrical double layer theory, the relationship between electrical charge and electrical potential at the slipping plane is given by:[2]

$$\boldsymbol{Q_e = 4\pi\varepsilon_0\varepsilon_r a(1 + \kappa a)\zeta} \quad \textbf{(1)}$$

where Q_e is the electrical charge at the slipping plane (the electrokinetic charge), ζ is the electrokinetic (zeta) potential which, by definition, is the electrical potential at the slipping plane, $\boldsymbol{\varepsilon_0}$ is the absolute permittivity in vacuo, $\boldsymbol{\varepsilon_r}$ is the relative permittivity (or dielectric constant) of the fluid, $\boldsymbol{a}$ is the particle radius (which is the distance between the particle's true surface and the slipping plane), and $\boldsymbol{\kappa}$ is the Debye-Hückel parameter, as given by:

$$\boldsymbol{\kappa = \left(\frac{2000F^2I}{\varepsilon_0\varepsilon_r RT}\right)^{1/2}} \quad \textbf{(2)}$$

where F is the Faraday constant, R is the ideal gas constant, T is the absolute temperature, and I is the ionic strength (mol dm^{-3}), as given by:

$$\boldsymbol{I = \frac{1}{2}\sum_i c_i z_i^2} \quad \textbf{(3)}$$

where c_i and z_i are the molar concentrations in bulk solution and the valency of ions of type i, respectively.

For a particle whose electrokinetic charge remains constant irrespective of the ionic composition of the bulk phase, Equation 1 indicates that there must be a reduction in zeta potential with increasing ionic strength. The apparent zeta potentials obtained for this study are comparable to those

determined for many types of particles dispersed in low ionic strength media (e.g. 0.001 M KCl). Given the hydrodynamic diameters reported above, Equations 2 and 3 indicate a κa value of approximately 6.5 to 8.5 in 0.001 M KCl. However, the ionic strength of the aqueous solution of 2.0 M ammonium sulfate used herein is approximately 6.1 M, hence the corresponding κa ranges from 510 to 663. Equation 1 predicts that the zeta potential at this relatively high ionic strength should be reduced to approximately 0.015 of its value at low ionic strength. This is not observed experimentally, which indicates that these particles cannot be described in terms of conventional electrokinetics. This classical theory assumes non-conducting hard spheres with smooth, impenetrable surfaces and a uniform charge distribution located entirely at the surface; it neither accounts for the finite size and hydration of ions at or near the particle surface nor for particles that are permeable towards the dispersion medium. Clearly, such assumptions are not valid for the particles studied herein. More specifically, the particles are best described as soft spheres and all the charged groups are located within the steric stabilizer layer, which is highly permeable to the surrounding liquid. The Hermans-Fujita model[3,4] considers the flow of liquid through a porous sphere. In the case of a polymer chain, liquid flow is determined by the forces arising from the interaction between the liquid and the monomer repeat units (or segments) when the polymer chain moves in response to an externally applied electric field (as is the case for a polyelectrolyte in solution during an electrophoresis experiment). This model predicts that the electrophoretic mobility for a non-free draining porous particle containing spatially fixed charges tends to a constant finite value with increasing ionic strength, whereas Equation 1 predicts that the electrophoretic mobility tends to zero for hard spheres. Indeed, this has been recently demonstrated experimentally for a globular protein (bovine serum albumin) in aqueous KCl solution using the NG-ELS technique.[2] Ohshima has considered the electrophoretic behavior of charged soft particles (*i.e.*, hard spheres coated with a weakly charged porous polymer layer) on a theoretical basis.[5] This theory incorporates some of the concepts within the Hermans-Fujita model and similarly predicts a non-zero

electrophoretic mobility at high ionic strength. The experimental data reported herein support this prediction.

The cationic $PATAC_{195}$-$PDMAC_{1000}$ and zwitterionic $PMPC_{139}$-$PDMAC_{1000}$ particles both exhibit a modest reduction in apparent zeta potential on addition of KOH that is not observed for the anionic $PAMPS_{250}$-$PDMAC_{1000}$ particles (see Figure 7). There is insufficient information available regarding the configurational properties of the steric stabilizer chains, including their permeability, to provide a satisfactory explanation for the observed reduction in the apparent zeta potential. As indicated above, liquid flow through a permeable network of charged polymer chains depends on the balance of various forces. Since the liquid contains dissolved ions, it is feasible that both the random translational diffusion of the particles and their electrophoretic mobility depend on the salt concentration. In the present study, ammonium sulfate was preferred over potassium sulfate or sodium sulfate because the former salt possesses significantly higher aqueous solubility. However, the ammonium cation complicates the solution chemistry owing to its buffering action, which involves an equilibrium between ammonium ions and neutral ammonia. Indeed, a distinct ammonia odor was noted at the end of each titration whereas the initial acidic dispersions were odorless.

References

1 R. Weast, *CRC Handbook of Chemistry and Physics*, CRC Press, Florida, 66th edn., 1986.

2 J. F. Miller, *Langmuir*, 2020, **36**, 8641–8654.

3 J. J. Hermans and Fujita H., *Proc. Koninkl. Ned. Akad. Wetenshap*, 1955, **B58**, 182.

4 J. J. Hermans, *J. Polym. Sci.*, 1955, **18**, 527–534.

5 H. Ohshima, *Electrophoresis*, 2006, **27**, 526–533.
